# Phytic Acid and Biochar: An Effective All Bio-Sourced Flame Retardant Formulation for Cotton Fabrics

**DOI:** 10.3390/polym12040811

**Published:** 2020-04-04

**Authors:** Marco Barbalini, Mattia Bartoli, Alberto Tagliaferro, Giulio Malucelli

**Affiliations:** 1 Department of Applied Science and Technology, and local INSTM Unit, Viale Teresa Michel 5, 15121 Alessandria, Italy; marco.barbalini@polito.it; 2 Department of Applied Science and Technology, C.so Duca degli Abruzzi 24, 10129 Torino, Italy; mattia.bartoli@polito.it (M.B.); alberto.tagliaferro@polito.it (A.T.)

**Keywords:** cotton, flame retardance, phytic acid, biochar, thermal stability, flammability tests, cone calorimetry tests, durability

## Abstract

Flame retardant systems based on bio-sourced products combine quite high fire performances with the low environmental impact related to their synthesis and exploitation. In this context, this work describes a new all bio-sourced flame retardant system designed and applied to cotton fabrics. In particular, it consists of phytic acid (PA), a phosphorus-based naturally occurring molecule extracted from different plant tissues, in combination with biochar (BC), a carbon-rich solid product obtained from the thermo-chemical conversion of biomasses in an oxygen-limited environment. PA and BC were mixed together at a 1:1 weight ratio in an aqueous medium, and applied to cotton at different loadings. As revealed by flammability and forced combustion tests, this bio-sourced system was able to provide significant improvements in flame retardance of cotton, even limiting the final dry add-on on the treated fabrics at 8 wt.% only. The so-treated fabrics were capable to achieve self-extinction in both horizontal and vertical flame spread tests; besides, they did not ignite under the exposure to 35 kW/m^2^ irradiative heat flux. Conversely, the proposed flame retardant treatment did not show a high washing fastness, though the washed flame retarded fabrics still exhibited a better flame retardant behavior than untreated cotton.

## 1. Introduction

The ease of flammability represents a major issue for cotton textiles, especially in those application fields, where fire-proofing is strictly required. In fact, when exposed to an irradiative heat flux or put in contact with a flame, this cellulosic material is easily ignited and the combustion takes place with high burning rate, leaving a negligible residue. In order to overcome this limitation, cotton needs to be effectively flame retarded, i.e., treated with specific chemical products intended to minimize the rate of flame spread and to inhibit sustained combustion [1,2,3,4].

For this purpose, over the last 50 years, different flame retardants (FRs) have been conceived, synthesized and applied to cotton, achieving high fire performances for the treated fibers and fabrics and therefore overcoming the above-mentioned problem. Among the different flame retardant systems for cotton cellulosic textiles, from halogenated products (mainly based on chlorinated or brominated structures), showing a high efficiency, but, at the same time, a high environmental impact and in some cases, toxicity, persistency, bioaccumulation [5], both the academics and industrial companies have addressed the research towards the development of safer products. This was achieved first with the design of flame retardants based on the phosphorus (alone or in combination with nitrogen) chemistry [6,7,8], thus obtaining very efficient and more green FR additives; then, more recently, nanofiller-based systems have been developed and successfully exploited [4,9].

Aiming at further designing and utilizing products with lower environmental impact, in the last decade several scientific papers started to investigate the big potentialities of biomacromolecules and bio-sourced products like proteins, nucleic acids, pomegranate rind extracts, banana pseudostem sap, among a few to mention, as effective flame retardant for either natural (i.e., cellulosic) or synthetic (mainly polyester) textiles [10,11,12,13,14,15].

These products possess three main advantages: first, their chemical structure and composition is very suitable for conferring flame retardant features to fabrics, as biomacromolecules and bio-sourced products contain some key elements (namely, phosphorus, nitrogen, sulfur), which are responsible for the activation of the flame retardant mechanisms [16]. Then, they are usually easily dispersible or soluble in water: this represents an advantage, as it bans the use of organic solvents with high environmental impact or even high toxicity. Finally, the FR treatments on the fabrics can be performed using the already available finishing units (i.e., industrial impregnation/exhaustion plants).

Specifically referring to phosphorus-based biomacromolecules, these FR additives usually exploit a condensed phase mechanism, assisting the formation of stable aromatic char [10,11]: this is possible because of the creation of phosphoric acids species upon the activation of the biomacromolecule, which favor dehydration reactions on the underlying textile substrate, remarkably limiting the production of organic flammable gases that can further fuel the combustion process. This mechanism is further enhanced when P-containing biomacromolecules are combined with a carbon source [17,18], as proposed in the present work.

In particular, here we take advantage from a new flame retardant “green” recipe that exploits the combination of phytic acid, a naturally occurring molecule, extracted from different plant tissues (e.g., oil seeds, soy beans and cereal grains [16]) and bearing six phosphate groups (28 wt.% of phosphorus based on molecular weight), with biochar, a carbon-rich solid product obtained from the thermo-chemical conversion of biomasses in an oxygen-limited environment [19,20].

Biochar, in particular, represents a very good carbon source that can be effectively exploited during the flame retardant action in condensed phase for enhancing the formation of a stable protective char on the burning cellulosic substrate [21].

The scientific literature already reports some interesting works dealing with the application of phytic acid-based flame retardants to different types of textile materials. In particular, this eco-friendly, biocompatible and nontoxic organic polyphosphoric acid has been successfully utilized as flame retardant for wool [22], silk [23], poly(lactic acid) nonwoven fabrics [24] and, more recently, cotton fabrics [25,26,27,28].

To the best of our knowledge, the flame retardant effects provided by the proposed all-bio-sourced system to cotton fabrics have not been investigated so far.

Therefore, first we combined PA and BC, keeping 1:1 weight ratio, in an aqueous medium, and applied to cotton at different loadings (from 4 to 10 wt.%), aiming to identify the best flame retardant treatment with the lowest dry add-on. The morphology of cotton and of the treated fabrics was investigated by means of SEM and FTIR-attenuated total reflection (ATR) spectroscopy analyses. As revealed by flame spread tests performed either in vertical or horizontal configuration on the different treated fabrics, it was possible to provide self-extinction to the cellulosic substrate limiting the final dry FR add-on to 8 wt.% only. This loading was also responsible for impeding the ignition of the fabrics under the cone calorimeter, using an irradiative heat flux of 35 kW/m^2^. Then, this most FR performing system in flame spread tests was further investigated as far as its thermal and thermo-oxidative behavior is considered and compared to that of untreated cotton and of the fabrics treated with BC or PA only. Finally, the durability (i.e., washing fastness) of the proposed flame retardant treatment was evaluated, comparing the flame retardance of the washed treated fabrics with the pristine counterparts.

## 2. Materials and Methods

### 2.1. Materials

Cotton fabrics (220 g/m^2^ and 0.2 mm thick) were purchased from Fratelli Ballesio S.r.l. (Torino, Italy).

Phytic acid (PA, as 50 wt.% aqueous solution) was purchased from Tokyo Chemical Industry Co., Ltd. (Oxford, United Kingdom) and used as received.

Ultra-pure 18.2 MU deionized water was supplied by a Q20 Millipore system (Milano, Italy).

Exhausted coffee powder collected from Bar Katia (Turin, Italy) and supplied by Vergnano Spa (Torino, Italy) as an Arabica mixture was employed as raw material for the preparation of Biochar. More specifically, the exhausted coffee was collected and dried at 105 °C for 72 h. Coffee samples (100 g) were pyrolyzed using a vertical furnace and a quartz reactor (heating rate: 15 °C/min) and kept at 800 °C for 30 min in argon atmosphere [19,20].

### 2.2. Preparation of Phytic Acid/Biochar Dispersions

The dispersions were prepared by mixing phytic acid and biochar at a 1:1 weight ratio. The mixtures were diluted under vigorous mechanical stirring with distilled water, in order either to promote the dispersion of the biochar into the aqueous solution, or to tune the final dry add-on on the cellulosic substrate.

### 2.3. Application of Phytic acid/Biochar Dispersions to Cotton Fabrics

Cotton fabrics were cut into square pieces (10 cm × 10 cm), weighted and then impregnated with the dispersions. The impregnated fabrics were put on a glass substrate and dried in an oven at 80 °C for 20 min. Then, the final dry add-on on the cotton samples (i.e., the dry weight gain (A%), wt.%) was determined by weighing each sample before (Wi) and after the impregnation with PA-BC dispersion and the subsequent thermal treatment (Wf). The weight gain was calculated using the following formula:

$$A\%=\frac{Wf-Wi}{Wi}*100$$

For a first set of impregnations, the dry add-on values were varied between 4 and 10 wt.%; furthermore, two cotton fabrics were treated with phytic acid (sample name: COT + PA) or biochar only (sample name: COT + BC, Table 1), achieving 8 wt.% dry add-on.

Table 1 lists the investigated composition, the wet pickup values and the different dry add-ons on the treated fabrics.

### 2.4. Characterization Techniques

A Perkin Elmer Spectrum 100 IR spectrometer equipped with an attenuated total reflection (ATR) diamond accessory was employed for collecting the FTIR-ATR spectra of untreated and treated cotton fabrics. FTIR-ATR spectra were recorded at wavelengths from 500 to 4000 cm^−1^ with 4 cm^−1^ resolution; for each specimen, 16 scans were collected.

A LEO-1450VP Scanning Electron Microscope (Zeiss, New Jersey, USA; beam voltage: 5 kV), coupled to an energy dispersive X-ray (EDX) micro-analyzer (mod. INCA Energy 300, Oxford instruments, Abingdon, UK) was employed to study the surface morphology of both untreated and treated samples. For the analysis, cotton fabric pieces (0.5 cm × 0.5 cm) were cut and fixed to conductive adhesive tapes and gold-metalized.

The thermal and thermo-oxidative stability of the fabrics was evaluated by thermogravimetric (TG) analyses in nitrogen and air, respectively, from 50 to 700 °C with a heating rate of 20 °C/min. To this aim, a TAQ500 analyzer (TA Instrument Inc., Waters LLC, DE, USA) was used, placing the samples (approximately 8 mg) in open alumina pans, in inert or oxidative atmosphere (gas flow: 35 mL/min).

Horizontal and vertical flame spread tests were carried out on untreated cotton and on treated fabrics according to UL94 standard.

Cone calorimetry tests were performed according to the ISO 5660 standard. More specifically, square specimens (10 cm × 10 cm) were irradiated with a heat flux of 35 (raised to 50 kW/m^2^, when ignition at 35 kW/m^2^ did not occur) in horizontal configuration; the fabrics were placed on a sample holder and maintained in the correct position using a metallic grid. For each formulation, the test was repeated three times and the results averaged. A standard deviation of 2% was calculated for the following parameters: Time to Ignition (TTI, s), Total Heat Release (THR, kW/m^2^), peak of Heal Release Rate (pkHRR, kW/m^2^). The residues at the end of the tests were also evaluated.

Finally, the washing fastness of the treated fabrics was determined following the AATCC test method 61 (2A)-1996 in the presence of a non-ionic detergent at 38 ± 3 °C.

## 3. Results and Discussion

### 3.1. FTIR-ATR Spectroscopy

**Figure 1:**
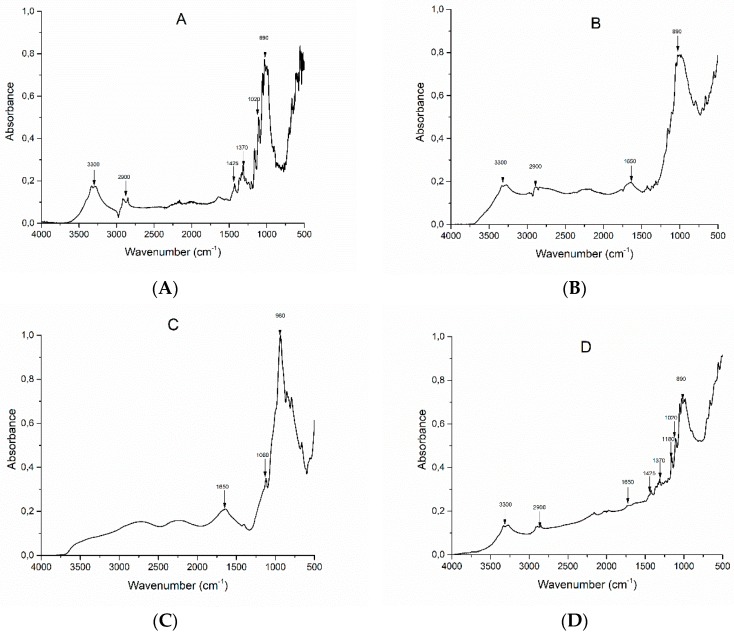
FTIR-attenuated total reflection (ATR) spectra of cotton (COT) (**A**), cotton+phytic acid (COT+PA) (**B**), PA (**C**) and cotton+phytic acid+biochar (COT+PA+BC(8)) (**D**).

The effectiveness of the deposition of the coatings on the cotton fabrics has been assessed through FTIR-ATR spectroscopy. Figure 1A–D compares the FT-IR spectra of untreated cotton, cotton treated with PA, pure PA and COT+PA+BC(8). Figure 1A shows the characteristic peaks of cellulose for untreated cotton (namely: *v*(OH) at ca. 3300 cm^−1^, *v*(CH_2_) at 2900 cm^−1^, δ (OH) at 1640 cm^−1^, δ (CH_2_) at 1425 cm^−1^, δ (CH) at 1370 cm^−1^, δ (OH) at 1310 cm^−1^, *v*(C–C) at 1020 cm^−1^ and δ (OH) at 894 cm^−1^) [29]. Figure 1C shows the FTIR-ATR spectrum of phytic acid: three characteristic peaks, located at 1650, 1060 and 980 cm^−1^ and corresponding to stretching vibration of P=O, asymmetric and symmetric stretching of P–O–C, are present [29]. These peaks are still detectable in COT+PA (Figure 1C).

In addition, the spectrum of cotton treated with phytic acid and biochar (Figure 1D) still shows the presence of some typical vibrational modes of cellulose, though these signals are less intense and defined because of the application of the flame retardant treatment.

### 3.2. Morphology of the Treated Fabrics

**Figure 2:**
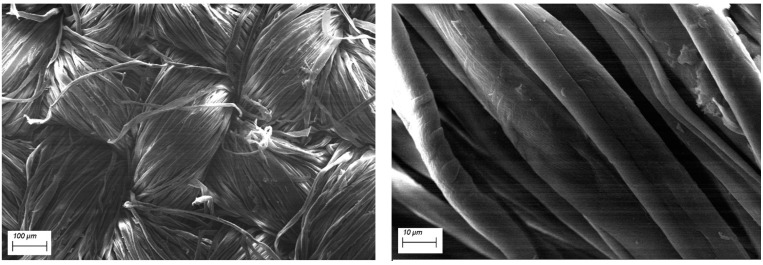
SEM images of untreated cotton at different magnifications.

**Figure 3:**
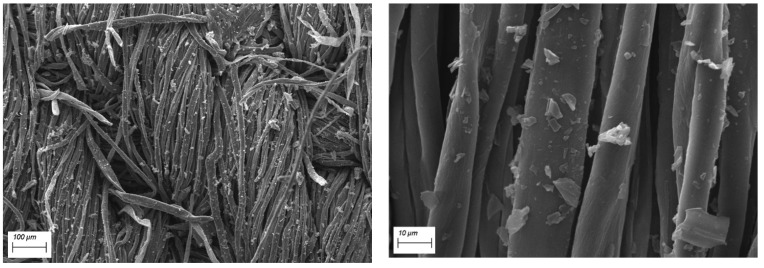
SEM images of COT+PA+BC(8) at different magnifications.

**Figure 4:**
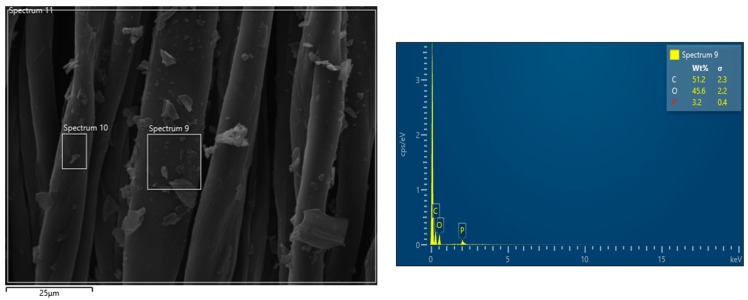
Energy dispersive X-ray (EDX) mapping of COT+PA+BC(8).

SEM-EDX observations have been performed in order to assess the morphology of the cotton fabrics before and after the application of the flame retardant treatment and to assess the presence of the flame retardant. Pure cotton is characterized by a quite smooth texture as evidenced in Figure 2, while the treated cotton fabrics (Figure 3 presents the typical morphology of COT + PA + BC(8) sample) show the appearance of some micro-sized BC particles on the surface. The elemental analysis carried out by energy dispersive X-ray spectroscopy on some areas where BC particles appear (Figure 4) confirms the presence of C, O and P elements (i.e., the main phytic acid and BC constituents, obviously not excluding the C and O contribution from the cellulosic substrate). It is noteworthy that PA, as already observed in sol-gel systems containing this bio-sourced product [27], is likely to coat the fibers with a homogeneous layer; BC micro-sized particles are quite well distributed on the fiber surface.

### 3.3. Flame Spread Tests

Flame spread tests, carried out either in vertical and horizontal configuration on cotton and on the different treated fabrics were exploited for a preliminary screening of the flame retardant properties, trying to identify the lowest final dry add-on that ensured self-extinction in both vertical and horizontal tests.

**Figure 5:**
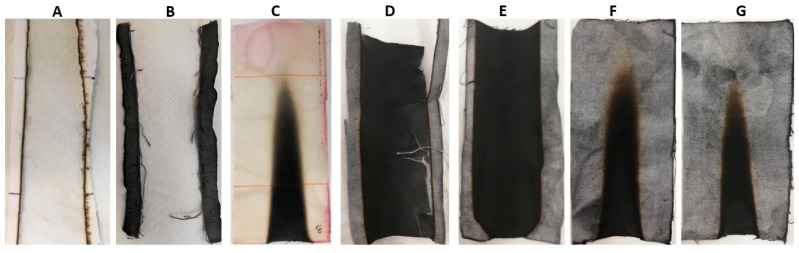
Residues after vertical flame spread tests. (**A**) COT, (**B**) COT+BC, (**C**) COT+PA, (**D**) COT+PA+BC(4), (**E**) COT+PA+BC(6), (**F**) COT+PA+BC(8), (**G**) COT+PA+BC(10).

Table 2 lists the results of vertical flame spread tests. First of all, it is noteworthy that the treatment with BC alone is not able to provide any enhancement, as the treated fabrics are not classified; conversely, the deposition of the phytic acid coating (8 wt.% add-on) makes self-extinguishing the underlying fabric, significantly increasing, at the same time, the residue at the end of the tests (53%). This latter is further enhanced (up to 85%) by partly replacing PA with BC, keeping the same final dry add-on of 8 wt.% and still achieving self-extinction: this finding is a clear indication of the synergistic effect taking place between PA and BC, which, together, significantly intensify the char-forming effect of PA, leading to the creation of a very stable carbonaceous residue. In addition, it is noteworthy that, irrespective of the UL94 classification achieved (i.e., NC or V0), all the treated textiles at the end of vertical flame spread tests show dense and coherent residues, maintaining the texture of the pristine fabric (Figure 5).

**Figure 6:**
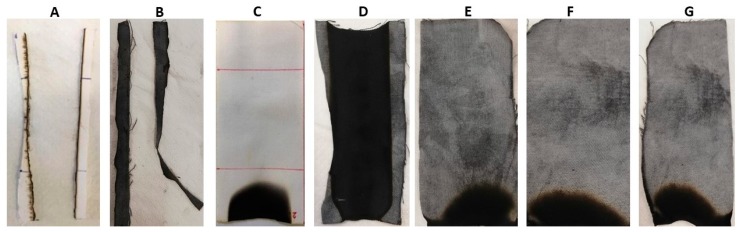
Residues after horizontal flame spread tests. (**A**) COT, (**B**) COT+BC, (**C**) COT+PA, (**D**) COT+PA+BC(4), (**E**) COT+PA+BC(6), (**F**) COT+PA+BC(8), (**G**) COT+PA+BC(10).

The results from vertical flammability tests are further confirmed by those obtained in the horizontal configuration: the latter are collected in Table 3. In addition, Figure 6 shows the typical images of the residues at the end of the tests: again, the residues are very coherent and the burnt part of the specimens still keeps the texture of the fabric, hence indicating a good protection exerted by the flame retardant treatment on the underlying cellulosic substrate.

### 3.4. Cone Calorimetry Tests

**Figure 7:**
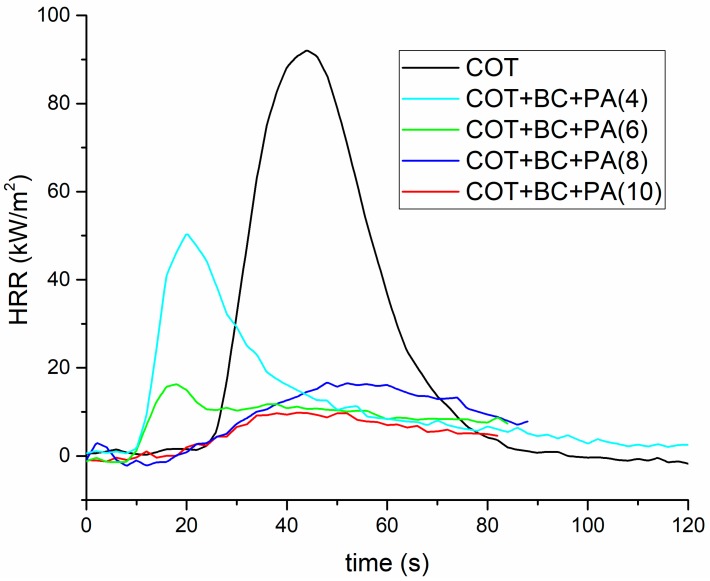
Heal Release Rate (HRR) vs. time curves for treated and untreated cotton fabrics (heat flux: 35 kW/m^2^).

In order to further support the flammability data discussed in the previous paragraph, forced combustion tests were performed on cotton and on the fabrics treated with the PA/BC flame retardant coating. The obtained data are listed in Table 4. Figure 7 shows the typical HRR curves vs. time. First of all, it is worthy to note that, when exposed to 35 kW/m^2^ irradiative heat flux, the only sample to ignite was COT+PA+BC(4); because of the activation of PA before the starting of the degradation of cotton, TTI is anticipated as compared to the untreated fabric. Furthermore, HRR, pkHRR and THR values of the FR-treated sample are significantly reduced and the final residue (10.4%) is remarkably increased: all these findings are a clear indication of the formation of a stable char, which protects the underlying fabric during the exposure to the heat flux. Conversely, all the specimens with a dry add-on of 6 wt.% or higher, do not ignite at 35 kW/m^2^, leaving, at the end of the tests, very high residues ranging within 22 and 25%. This finding clearly indicates that the concurrent presence of PA and BC in the flame retardant coating shows synergistic effects occurring between the two constituents.

**Figure 8:**
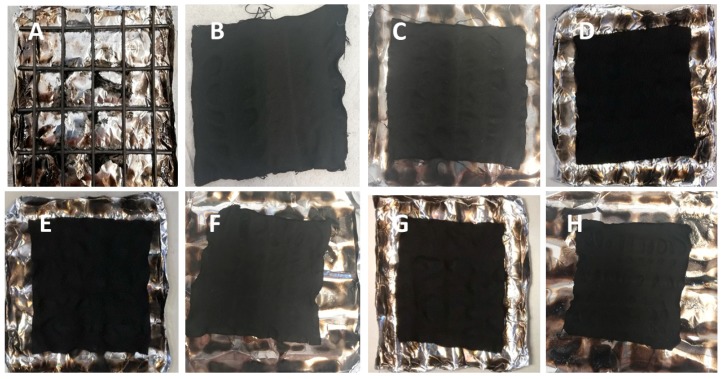
Residues of COT (**A**), COT+PA+BC(10) (**B**), COT+PA+BC(8) (**C**), COT+PA+BC(6) (**D**) and COT+PA+BC(4) (**E**) after cone calorimetry tests performed at 35 kW/m^2^. Residues of COT+PA+BC(10) (**F**), COT+PA+BC(8) (**G**) and COT+PA+BC(6) (**H**) after cone calorimetry tests performed at 50 kW/m^2^.

Table 4 also collects the forced combustion data obtained at 50 kW/m^2^. It is worthy to highlight that at this irradiative heat flux the flame retarded fabrics ignite; in addition, the decrease of HRR, pkHRR and THR values, and the rise of the residues as well, are strictly related to the increase of the dry add-on. Finally, some typical images of the residues after forced combustion tests performed at the two irradiative heat fluxes are shown in Figure 8.

### 3.5. Thermal And Thermo-Oxidative Stability of The Treated Fabrics

**Figure 9:**
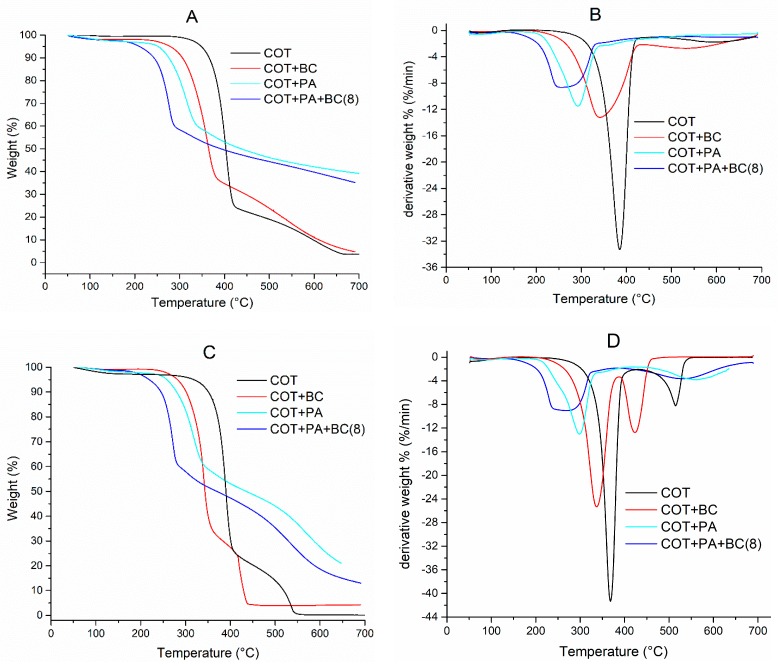
Thermogravimetric (TG) and dTG curves in N_2_ (**A**,**B**) and air (**C**,**D**) of untreated and treated cotton fabrics.

Thermogravimetric (TG) analyses have been utilized for evaluating the thermal and thermo-oxidative stability of untreated cotton and the fabric treated with PA or BC alone, or with the best combination of the two, having the lowest dry add-on that provided self-extinction in flame spread tests (i.e., COT+PA+BC(8)). T_ONSET_, the maximum weight loss temperatures (T_max1_ and T_max2_), the corresponding residues and the final residue at 700 °C are listed in Table 5. Figure 9A–D shows the typical TG and dTG curves.

In nitrogen atmosphere, the decomposition of untreated cotton takes place according to a single main degradation step; the degradation onset occurs at about 358 °C and the maximum degradation rate is observed at 386 °C (Table 5). The presence of PA or BC or their combination on the fabric substrate is responsible for an anticipation of the cellulose decomposition temperature as revealed by T_max_ and T_ONSET_, which are shifted towards lower values (i.e., 270, 311 and 250 °C, for COT+PA, COT+BC and COT+PA+BC(8). These shifts, as already assessed in sol-gel derived systems containing PA [30], are more evident when phytic acid is present in the flame retardant formulation, as it starts decomposing prior to the decomposition of the cellulosic substrate. This finding has been already found for cotton fabrics treated with selected biomacromolecules [10]: when the flame retardant coating activates, it favors the formation of a stable char, able to behave as a thermal barrier, in place of the formation of combustible gaseous products that could further stimulate the degradation of the cellulosic material [20]. In addition, the residues at 700 °C for all the fabrics treated with the formulations containing PA are much higher with respect to that obtained for untreated cotton, again confirming the protection exerted by the deposited PA-containing coatings on the underlying fabric.

In air, cotton decomposition occurs by two steps. The first (dTG peak at 368 °C, Table 5) involves two competitive pathways, which produce aliphatic char and volatile products; during the second step (dTG peak at 514 °C, Table 5), the aliphatic char is converted into an aromatic form, producing CO and CO_2_ as a consequence of simultaneous carbonization and char oxidation [21].

As already observed in an N_2_ atmosphere, the flame retardant treatment anticipates the decomposition of the cellulosic substrate: in fact, T_ONSET_ and T_max1_ values are shifted towards lower temperatures as compared to untreated cotton. At the same time, a stable char is formed, as revealed by the residues calculated at T_max2_ and 700 °C, which remarkably increase when cotton is treated with FR finishing containing PA, alone or in combination with BC. Once again, this behavior is ascribable to the activation of phytic acid, which decomposes prior the cellulosic substrate, favoring dehydration reactions on the fabric to take place, thus forming a protective stable char layer, limiting, at the same time, the development of combustible gaseous species.

### 3.6. Durability of the Designed FR Treatments

**Figure 10:**
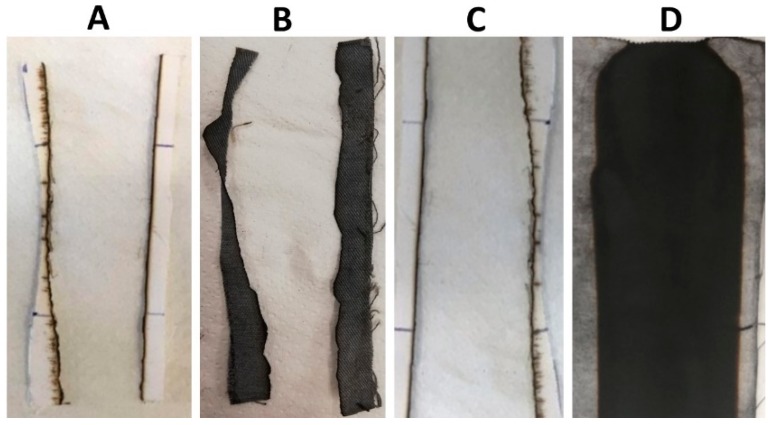
Residues after vertical flame spread tests performed on washed fabrics. (**A**) COT, (**B**) COT+PA W, (**C**) COT+BC W, (**D**) COT+PA+BC(8) W.

For many application fields, the washing fastness of the flame retarded fabrics is an important issue that can significantly limit their practical use. Therefore, some tests for evaluating the durability were performed according to the AATCC test method 61 (2A)-1996 on selected treated fabrics; then, the fabrics were subjected to horizontal flame spread tests: the obtained data are collected in Table 6, while Figure 10 shows the residues at the end of the tests.

The obtained results clearly indicate that the FR treated fabrics have poor resistance to washing cycles: in fact, even the fabrics that achieved self-extinction (see Table 3), lose this feature after laundry. However, after washing, COT+PA+BC(8) (coded as COT+PA+BC(8) W in Table 6) still performs better with respect to untreated cotton, leaving a coherent residue at the end of the burning test (Figure 10D).

**Figure 11:**
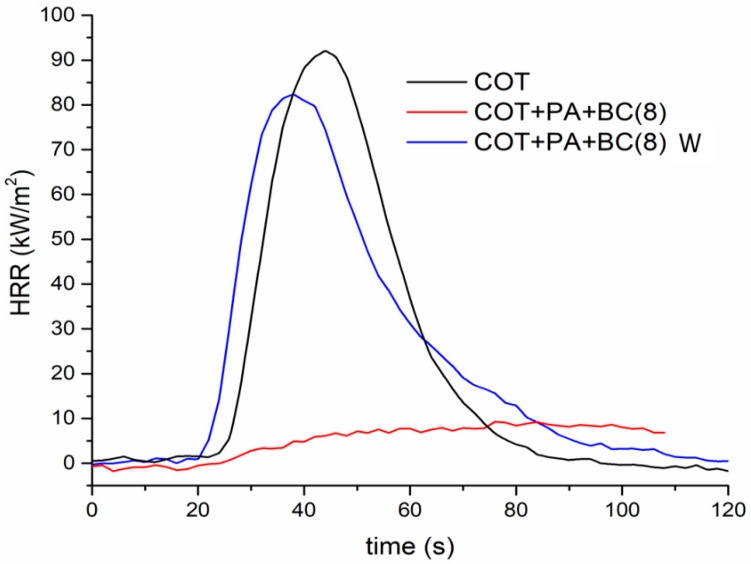
HRR vs. time curves for cotton and for COT+PA+BC(8) before and after washing (heat flux: 35 kW/m^2^).

**Figure 12:**
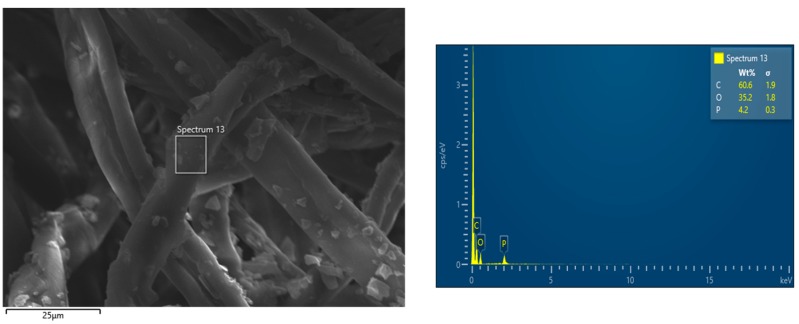
EDX mapping of the residue of COT+PA+BC(8) W after horizontal flame spread tests.

**Figure 13:**
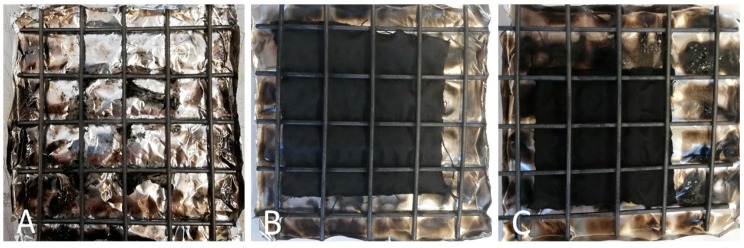
Residues of COT (**A**), COT+PA+BC(8) (**B**) and COT+PA+BC(8)W (**C**) after cone calorimetry tests (heat flux of 35 kW/m^2^).

Furthermore, Table 7 compares the forced-combustion data for COT, COT+PA+BC(8) and COT+PA+BC(8)W (i.e., the best performing sample before and after washing cycles, respectively). Figure 11 shows the corresponding HRR vs. time curves. The significant loss of the flame retardant coating as a consequence of the washing cycles allows the sample to ignite at 35 kW/m^2^ irradiative heat flux; however, the thermal parameters of the washed sample are still better than those of untreated cotton, because of the effect of the FR coating that resisted to the washing process (see EDX analysis, shown in Figure 12, of the residue after horizontal flame spread tests of COT+PA+BC(8) W; the residues after cone calorimetry tests are displayed in Figure 13).

The removal of flame retardant was reasonably due to the weak interactions between BC and the cotton fabrics represented by hydrogen-like bonds between hydroxylic functionalities of cotton and π−orbital systems of BC graphitic domains. As observed by Levitt et al. [30], these interactions are weaker than typical hydrogen bonds, with a maximum value close to 21 kJ/mol: therefore, a simple washing treatment can easily remove most of the coating from the underlying cellulosic substrate.

## 4. Conclusions

In this work, a novel all bio-sourced flame retardant waterborne system comprising phytic acid and biochar was designed and applied to cotton fabrics. The combination of the two components allowed developing flame retardant synergistic effects on the cellulosic substrate. In particular, flame spread tests showed that it was possible to achieve self-extinction with a very limited final dry add-on (i.e., 8 wt.%) on the fabrics. Furthermore, in forced-combustion tests, no ignition was observed for the fabrics treated with both PA and BC at 8 wt.% add-on, when exposed to 35 kW/m^2^ irradiative heat flux. Conversely, the washing fastness of the treated fabrics was not acceptable, as the FR features were significantly lost after washing cycles, though the washed flame retarded fabrics still exhibited higher flame retardant features with respect to untreated cotton. Therefore, future research work will be devoted to improve the durability of the proposed treatment, trying to maintain either a low environmental impact or the bio-source characteristics of the new modified flame retardant coatings.

## Figures and Tables

**Table 1:** Composition of the treated cotton fabrics investigated.

Sample Code	PA (wt.%)	BC (wt.%)	Wet Pickup (wt.%)	Dry Add-On A% (wt.%)
COT+PA	100	0	78	8
COT+BC	0	100	84	8
COT+PA+BC(4)	50	50	85	4
COT+PA+BC(6)	50	50	86	6
COT+PA+BC(8)	50	50	87	8
COT+PA+BC(10)	50	50	87	10

**Table 2:** Results of vertical flame spread tests performed on cotton and on the treated fabrics.

SAMPLE	Total Dry Add-On (%)	SELF-EXTINCTION	Residue (%)	Classification
COT	/	NO	0	NC
COT+BC	8	NO	0	NC
COT+PA	8	YES	53	V0
COT+PA+BC(4)	4	NO	51	NC
COT+PA+BC(6)	6	NO	55	NC
COT+PA+BC(8)	8	YES	85	V0
COT+PA+BC(10)	10	YES	90	V0

**Table 3:** Results of horizontal flame spread tests performed on cotton and on the treated fabrics.

SAMPLE	Total Add-On (%)	t_1_ (s)	t_2_ (s)	t_tot_ (s)	Burning Rate (mm/s)	Residue (%)
COT	/	12	40	63	1.58	0
COT+BC	8	13	41	63	1.58	0
COT+PA	8	/	/	/	/	92
COT+PA+BC(4)	4	15	45	70	1.42	57
COT+PA+BC(6)	6	/	/	/	/	95
COT+PA+BC(8)	8	/	/	/	/	97
COT+PA+BC(10)	10	/	/	/	/	97

**Table 4:** Forced-combustion data for untreated and treated cotton fabrics.

**Sample**	**Time to Ignition (s)**	**HRR (kW/m^2^)**	**pkHRR (kW/m^2^)**	**Time to Peak (s)**	**THR (MJ/m^2^)**	**Residue** **(%)**
**Heat flux: 35 kW/m^2^**						
COT	19	15.6	96	38	2.0	0
COT+PA+BC(4)	10	12.4	53	20	1.5	10.4
COT+PA+BC(6)	No ignition					22.1
COT+PA+BC(8)	No ignition					24.5
COT+PA+BC(10)	No ignition					24.7
**Heat flux: 50 kW/m^2^**						
COT	15	16.9	105	32	2.1	0
COT+PA+BC(6)	7	14.3	65	20	2.0	12.5
COT+PA+BC(8)	7	11.7	56	16	1.9	15.5
COT+PA+BC(10)	6	10.9	53	15	1.2	18.7

**Table 5:** Results from thermogravimetric analyses in nitrogen and air for cotton and for the treated fabrics.

Sample Code	T_ONSET_ (°C)	T_max1_ (°C)	Residue @T_max1_ (%)	T_max2_ (°C)	Residue @T_max2_ (%)	Residue @ 700 °C (%)
*Atmosphere: nitrogen*						
COT	358	386	48.3	-	-	3.8
COT+PA	270	290	75.0	-	-	37.0
COT+BC	311	341	71.0	-	-	4.9
COT+PA+BC(8)	250	241	89.1	-	-	36.0
*Atmosphere: air*						
COT	350	368	53.0	514	7.3	3.5
COT+PA	265	296	73.0	550	30.2	20.0
COT+BC	321	337	65.5	425	14.0	3.9
COT+PA+BC(8)	248	263	80.2	540	28.5	13.1

**Table 6:** Horizontal flame spread tests performed on cotton and on selected flame retardant (FR) treated samples after washing.

SAMPLE	Dry Add-On (%)	t_1_ (s)	t_2_ (s)	t_tot_ (s)	Burning Rate (mm/s)	Residue (%)
COT	/	12	40	63	1.58	0
COT+PA W	8	13	44	69	1.44	2
COT+BC W	8	13	43	64	1.56	0
COT+PA+BC(8) W	8	15	48	75	1.33	18

**Table 7:** Combustion data for untreated and treated cotton fabrics after washing (heat flux: 35 kW/m^2^).

Sample	Time to Ignition (s)	HRR (kW/m^2^)	pkHRR (kW/m^2^)	Time to Peak (s)	THR (MJ/m^2^)	Residue (%)
COT	19	15.6	96.4	38	2.0	0
COT+PA+BC(8)	No ignition					24.5
COT+PA+BC(8) W	10.5	14.8	80.9	22	1.6	12.5
